# Supplementary material for: Intricate Knots in Proteins: Function and Evolution
Source: PLoS Comput Biol. 2006 Sep 15;2(9):e122. doi: 10.1371/journal.pcbi.0020122 (PMC1570178; doi:10.1371/journal.pcbi.0020122)
Supplement: Supplementary file 1 [file pcbi.0020122.st001.doc]

In the following we provide a list of 273 knotted entries from which table 1 was extracted:

| 12ca | 1a42 | 2abe | 1aiv | 1ajc | 1am6 | 1aov | 1avn | 2ax2 | 1azm | 2b63 | 1b90 | 1bcd | 5bca | 1bic |
| --- | --- | --- | --- | --- | --- | --- | --- | --- | --- | --- | --- | --- | --- | --- |
| 1bn1 | 1bn3 | 1bn4 | 1bnm | 1bnn | 1bnq | 1bnt | 1bnu | 1bnv | 1bnw | 1bv3 | 1bzm | 1c5m | 1ca2 | 1ca3 |
| 1cah | 1cai | 1caj | 1cak | 1cal | 1cam | 1can | 1cao | 1cay | 1caz | 2ca2 | 2cab | 3ca2 | 4ca2 | 4cac |
| 5ca2 | 5cac | 6ca2 | 7ca2 | 8ca2 | 9ca2 | 2cba | 2cbb | 2cbc | 2cbd | 2cbe | 1ccs | 1cct | 1ccu | 1cil |
| 1cim | 1cin | 1cnb | 1cnc | 1cng | 1cnh | 1cni | 1cnj | 1cnk | 1cnw | 1cnx | 1cny | 1cqx | 1cra | 1crm |
| 1cva | 1cvb | 1cvc | 1cvd | 1cve | 1cvf | 1cvh | 2cx8 | 1czm | 1dca | 1dcb | 1dmx | 1dmy | 1dqu | 1eou |
| 1f2w | 1flj | 1fnm | 1fql | 1fqm | 1fqn | 1fqr | 1fr4 | 1fr7 | 1fsn | 1fsq | 1fsr | 1fug | 1g0e | 1g0f |
| 1g1d | 1g3z | 1g45 | 1g46 | 1g48 | 1g4j | 1g4o | 1g52 | 1g53 | 1g54 | 1g6v | 1gz0 | 1h4n | 2h4n | 1h9n |
| 1h9q | 1hca | 1hcb | 1hea | 1heb | 1hec | 1hed | 1hug | 1huh | 1hva | 1hys | 1i50 | 1i6h | 1i8z | 1i90 |
| 1i91 | 1i9l | 1i9m | 1i9n | 1i9o | 1i9p | 1i9q | 1if4 | 1if5 | 1if6 | 1if7 | 1if8 | 1if9 | 1ipa | 1itc |
| 1j0y | 1j0z | 1j10 | 1j11 | 1j12 | 1j2o | 1j85 | 1j9w | 1jcz | 1jd0 | 1js1 | 1jv0 | 1jzu | 1k3r | 1keq |
| 1kop | 1koq | 1kwq | 1kwr | 1lg5 | 1lg6 | 1lgd | 1lug | 1lzv | 1moo | 1mua | 1mxi | 1nik | 1ns5 | 1nt9 |
| 1nxz | 1o6d | 1okl | 1okm | 1okn | 1oq5 | 1osn | 1ovt | 1p35 | 1p73 | 1p75 | 1p7c | 1p7l | 1p9p | 1pmd |
| 1poq | 1qmg | 1r9s | 1r9t | 1ray | 1raz | 1rg9 | 1rj5 | 1rj6 | 1rkn | 1rza | 1rzb | 1rzc | 1rzd | 1rze |
| 1s1h | 1sfo | 1t9n | 1tb0 | 1tbt | 1te3 | 1teq | 1teu | 1tg3 | 1tg9 | 1th9 | 1thk | 1to0 | 1ttm | 1uaj |
| 1uak | 1ual | 1uam | 1uga | 1ugb | 1ugc | 1ugd | 1uge | 1ugf | 1ugg | 1urt | 1v2x | 1v6z | 1v9e | 1v9i |
| 1vh0 | 1vhy | 1wcm | 1x7o | 1x7p | 1xd3 | 1xeg | 1xev | 1xpz | 1xq0 | 1y1w | 1y77 | 1y7w | 1y8a | 1yda |
| 1ydb | 1ydc | 1ydd | 1yh0 | 1yh1 | 1yo0 | 1yo1 | 1yo2 | 1yrl | 1yve | 1z93 | 1z97 | 1ze8 | 1zjr | 1znc |
| 1zsa | 1zsb | 1zsc |  |  |  |  |  |  |  |  |  |  |  |  |

**Table S1:** List of knotted pdb entries
