# Supplementary material for: Intricate Knots in Proteins: Function and Evolution
Source: PLoS Comput Biol. 2006 Sep 15;2(9):e122. doi: 10.1371/journal.pcbi.0020122 (PMC1570178; doi:10.1371/journal.pcbi.0020122)
Supplement: Supplementary file 2 [file pcbi.0020122.st002.doc]

The following 32 entries from table S1 become unknotted when the random closure method is applied (see Methods section):

| 1aiv | 1aov | 2b63 | 1b90 | 5bca | 1c5m | 1cqx | 1dqu | 1fnm | 1hys | 1i50 | 1i6h | 1itc | 1j0y | 1j0z |
| --- | --- | --- | --- | --- | --- | --- | --- | --- | --- | --- | --- | --- | --- | --- |
| 1j10 | 1j11 | 1j12 | 1jzu | 1nik | 1nt9 | 1ovt | 1p35 | 1pmd | 1r9s | 1r9t | 1rkn | 1sfo | 1wcm | 1y1w |
| 1y77 | 1y8a |  |  |  |  |  |  |  |  |  |  |  |  |  |

**Table S2:** List of knotted entries from S1, which become unknotted when ends are connected by the random closure method.
