# Supplementary material for: Intricate Knots in Proteins: Function and Evolution
Source: PLoS Comput Biol. 2006 Sep 15;2(9):e122. doi: 10.1371/journal.pcbi.0020122 (PMC1570178; doi:10.1371/journal.pcbi.0020122)
Supplement: Supplementary file 3 [file pcbi.0020122.st003.doc]

The following 75 structures become knotted only when missing sections are joined by straight lines:

| 2a0i | 1ath | 1bmf | 2bm0 | 2btv | 1by7 | 2c1g | 2c1i | 2cau | 2cav | 1cmx | 1cow | 1e1r | 1efr | 1eku |
| --- | --- | --- | --- | --- | --- | --- | --- | --- | --- | --- | --- | --- | --- | --- |
| 1ep4 | 1f48 | 1giy | 1gjj | 1gku | 1gqh | 1he8 | 1ii0 | 1ii9 | 1jqo | 1jr1 | 1juh | 1jwt | 1jxq | 1m1x |
| 1m72 | 1mas | 1ml5 | 1mxa | 1mxb | 1mxc | 1nbm | 1o6l | 1o90 | 1o92 | 1o93 | 1o9t | 1ova | 1phj | 2pps |
| 1pv2 | 1qm4 | 1qo1 | 1qx0 | 1qxp | 1rty | 1t0h | 1t0j | 1u2z | 1u7l | 1uch | 1uij | 1vhk | 1w0k | 1w5c |
| 1wd8 | 1xi4 | 1xi5 | 1xra | 1xrb | 1xrc | 1ybw | 1yc0 | 1yl3 | 1ypv | 1yvu | 1z85 | 2znc | 3znc | 1ztu |

**Table S3:** List of structures which become knotted when missing sections are joined by straight lines.
